# Construction and Analysis of Food-Grade *Lactobacillus kefiranofaciens* β-Galactosidase Overexpression System

**DOI:** 10.4014/jmb.2101.01028

**Published:** 2021-02-24

**Authors:** Xi He, MingJian Luan, Ning Han, Ting Wang, Xiangzhong Zhao, Yanyan Yao

**Affiliations:** 1 State Key Laboratory of Biobased Material and Green Papermaking, Qilu University of Technology, Shandong Academy of Sciences, Jinan, Shandong Province, P.R. China; 2 College of Biologic Engineering, Qi Lu University of Technology, Jinan, Shandong Province, P.R. China; 3 National Engineering Research Center for Marine Shellfish, Weihai, Shandong Province, P.R. China

**Keywords:** *Lactobacillus kefiranofaciens*, β-galactosidases, GOS, transgalactosylation, hydrolytic, food grade

## Abstract

*Lactobacillus kefiranofaciens* contains two types of β-galactosidase, LacLM and LacZ, belonging to different glycoside hydrolase families. The difference in function between them has been unclear so far for practical application. In this study, *LacLM* and *LacZ* from *L. kefiranofaciens* ATCC51647 were cloned into constitutive lactobacillal expression vector pMG36e, respectively. Furtherly, pMG36n-*lacs* was constructed from pMG36e-*lacs* by replacing erythromycin with nisin as selective marker for food-grade expressing systems in *Lactobacillus plantarum* WCFS1, designated recombinant LacLM and LacZ respectively. The results from hydrolysis of o-nitrophenyl-β-galactopyranoside (ONPG) showed that the β-galactosidases activity of the recombinant LacLM and LacZ was 1460% and 670% higher than that of the original *L. kefiranofaciens*. Moreover, the lactose hydrolytic activity of recombinant LacLM was higher than that of LacZ in milk. Nevertheless, compare to LacZ, in 25% lactose solution the galacto-oligosaccharides (GOS) production of recombinant LacLM was lower. Therefore, two β-galactopyranosides could play different roles in carbohydrate metabolism of *L. kefiranofaciens*. In addition, the maximal growth rate of two recombinant strains were evaluated with different temperature level and nisin concentration in fermentation assay for practical purpose. The results displayed that 37°C and 20-40 U/ml nisin were the optimal fermentation conditions for the growth of recombinant β-galactosidase strains. Altogether the food-grade Expression system of recombinant β-galactosidase was feasible for applications in the food and dairy industry.

## Introduction

As one of the probiotic LAB (lactic acid bacteria), *Lactobacillus kefiranofaciens* has been detected in fermented cow milk or goat milk from Tibet and the Caucasus region [1-3]. Moreover, *L. kefiranofaciens* generally localizes in kefir grains, natural starters for milk fermentation which composed of several fungal and bacterial species [4]. There are many advantages for this type of LAB. For example, *L. kefiranofaciens* can secrete antioxidant, antiulcer and antitumor, which can be used to enhance the immune system and lower blood cholesterol [5-7].

Another important advantage for *L. kefiranofaciens* is that it can yield β-galactosidases. β-Galactosidase (E.C. 3.2.1.23) catalyzes both the hydrolytic and transgalactosylation reactions of lactose, which are widely used in food, pharmaceutical and chemical industry [8, 9]. In terms of its hydrolytic function, β-galactosidase is added in milk to reduce the amount of lactose, keeping lactose intolerance group away from milk limiting [10, 11]. Furthermore, transgalactosylation reaction of β-galactosidase can produce so-called galacto-oligosaccharides (GOS), which have been widely used as ingredients in infant formula [12-14]. Up to now, according to the composition of amino acids, β-galactosidases have been categorized into four glycoside hydrolase (GH) families, including GH1, GH2, GH35 and GH42 (carbohydrate-active enzymes database; http://www.cazy.org) [15]. Interestingly, unlike other organisms containing only one type of β-galactosidase, some LAB such as *L. acidophilus* possess both GH2 and GH42 types of lactase, which named LacLM and LacZ respectively [16]. This phenomenon also exists in *L. kefiranofaciens*. Our previous study shows that the *lacLM* of *L. kefiranofaciens* belonging to GH2 family encodes a heterodimeric β-galactosidase, which consists of two overlapping genes, *lacL* and *lacM* encoding large and small subunits with calculated molecular mass of 73 kDa and 35 kDa (GI: 333957179, 333957180), respectively. In contrast, the LacZ gene belonging to GH42 family encodes one polypeptide chain with a calculated molecular mass of 76 kDa [17].

Many studies have focused on the β-galactosidase catalytic characteristic especially the ability to hydrolyze lactose and found it was common for LAB that LacLM type of β-galactosidase displays a strong ability to hydrolyze lactose [18, 19]. Xi *et al*. [17] cloned and overexpressed *lacLM* genes of *L. kefiranofaciens* ZW3 in *E. coli*, and then purified the recombinant LacLM to analysis the activity trait. The optimal ONPG hydrolysis activity for recombinant LacLM was observed at 55-65°C and pH 7.0-8.0. Moreover, our previous study also cloned and over-expressed *lacZ* gene of *L. kefiranofaciens* in *E. coli* and recombinant LacZ exhibited obviously lower hydrolytic activity than that of LacLM (Data are not shown).

Although the β-galactosidases gene of *L. kefiranofaciens* were cloned and overexpressed in *E. coli* successfully, while LAB, including *L. plantarum*, are increasingly considered as safe and attractive expression hosts and cell factories, especially for food-application purposes [20]. In addition, *L. plantarum* is suitable for large-scale industrial fermentation production. Nguyen *et al*. (2015) [20] inserted *lacLM* of *L. acidophilus* R22 into the lactobacillal expression vector pSIP403 and expressed in the host strains *L. plantarum* WCFS1. However, the pSIP403 vector uses erythromycin resistant gene as selective markers, so erythromycin must be added in fermentation broth to keep plasmid stability, which limits its application in the food industry. Thus, it is necessary to find another marker for food safety. Currently, the use of nisin as a selective marker for the transformed strain is recognized as effectivity and safety. Because nisin is a bacteriocin secreted from food grade *lactococcus lactis*, and has inhibitory effects on many bacteria as well, especially gram-positive bacteria. It is therefore a food safety additive approved by the FDA in 1969 [21]. Maischberger *et al*. (2010) [22] constructed a nisin-controlled gene expression (NICE) vector pTM and expressed *lacLM* from *Lactobacillus reuteri*, *L. acidophilus*, *L. sakei*, and *L. plantarum* in *Lactococcus lactis* hosts. But compared with *Lactococcus*, this approach has not always been straightforward or successful for *Lactobacilli* [20].

In this study, we took the constitutive-expression vector pMG36e as the object and transformed it into a food-grade expression vector. The pMG36e plasmid is derived from pVW01, which replicates in *Lactobacilli*, *Lactococcus*, *Bacillus subtilis*, and *Escherichia coli* [23]. Moreover, the vector contains a strong promoter P32, which high level of expression can be obtained without adding any inducing substance [24]. However, pMG36e uses erythromycin resistant gene as selective marker, which does not meet the needs of food safety. Follow these ideas two β-galactosidase genes *lacLM* and *lacZ* from *L. kefiranofaciens* ATCC51647 were cloned into pMG36e to construct expression vector pMG36e-*lac*. And then, pMG36n-*lacLM* and pMG36n-*lacZ* expression system were obtained by nisin resistant sequence *nisI* replacing original erythromycin resistant sequence *emr* in pMG36e, employing *L. plantarum* WCFS1 as a host strain. Furthermore, the hydrolysis activity of β-galactosidase and optimal growth conditions of the two recombinant strains were analyzed for the practices.

## Materials and Methods

### Cloning of *lacLM* and *lacZ* Genes

The *L. kefiranofaciens* ATCC51647 and *L. plantarum* WCFS1 strain (CGMCC, China) were grown in MRS at 37°C for 3 days. Then a Bacterial Gen DNA kit (Cowin, Beijing, China) was used to isolate the genomic DNA from *L. kefiranofaciens*. Specific primers were designed and synthesized (Table 1) according to the sequences of *lacLM* and *lacZ* in GenBank (Accession No. CP045033.1). Polymerase chain reaction (PCR) was used to amplify these genes from the genomic DNA. Briefly, after an initial denaturation step at 94°C for 5 min, amplifications were conducted with 35 cycles at 94°C for 30 sec, 62°C for 30 sec and 72°C for 2-3 min, followed by an extra extension step at 72°C for 10 min. The PCR products were isolated from agarose gels. The purified DNA fragments were ligated to pEASY-Blunt, and the plasmids were transformed into *E. coli* DH5α (Novagen, USA). The resulting recombinant plasmids were extracted from a positive clone and then sequenced.

### Construction the Recombinant Plasmid pMG36e-*lac*

One pair of restriction enzymes, SacI and HindIII, were used to excise the genes from the pEASY-Blunt recombinant plasmids, and then the genes were ligated to the pMG36e vector, which was digested with the same pair of restriction enzymes. Briefly, the ligation was carried out at 16°C overnight using T4 DNA ligase (Takara, China). *E. coli* DH5α competent cells were transformed with the ligation mixture and plated on Luria-Bertani (LB) agar containing erythromycin (400 μg/ml). The recombinant plasmids pMG36e-*lacLM* and pMG36e-*lacZ* were extracted by Plasmid Extraction Kit (Cowin, China) and identified by the SacI and HindIII enzyme digestion and PCR using specific primers (Table 1).

### Construction of pMG36n-lacs Plasmid

**Fig. 1:**
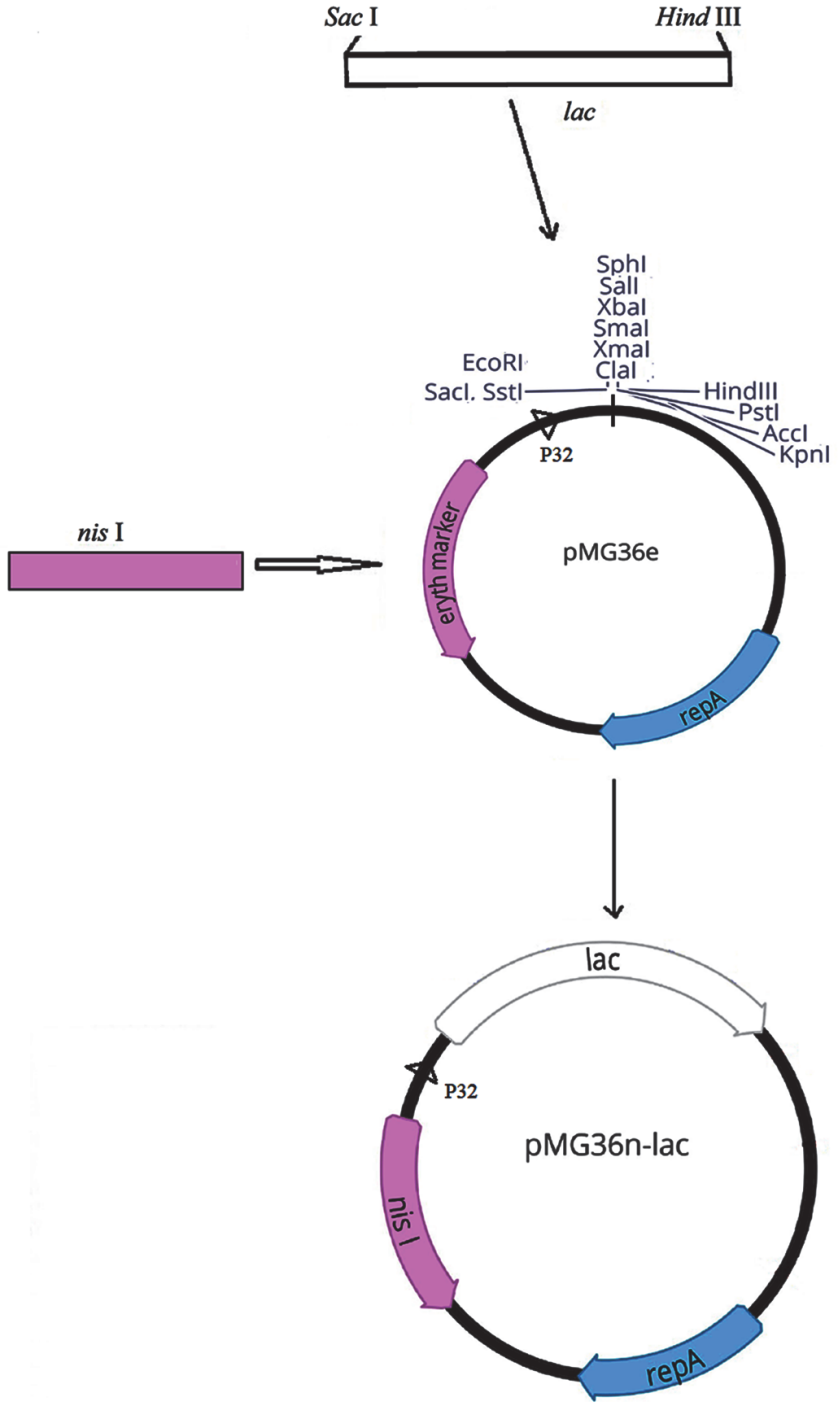
Vector map of pMG36n plasmids used in this study: lac, β-galactosidase structural gene; eryth marker, Erythromycin resistant marker; repA, replication determinants; P32, P32 promoter.

The *nisI* fragment was obtained from pet30a-*nisI* plasmid (Synthesized by Shanghai Personal Biotechnology Co., Ltd) by PCR with the 5′ and 3′ terminal containing homologous arm sequences identical to the pMG36e plasmid inserted site (Table 1). Similarly, the linear fragments without erythromycin resistant gene was acquired from the pMG36e-*lacLM* and pMG36e-*lacZ* plasmid by reverse PCR and the primers also were showed in Table 1. Then, the *nisI* fragment and the above linear fragments were homologous recombined by ClonExpress Ultra One Step Cloning kit (Vazyme, China). These new plasmids were designated as pMG36n-*lacLM* and pMG36n-*lacZ*, respectively (Fig. 1).

### Transformation in *L. plantarum*

The pMG36n-*lacLM* and pMG36n-*lacZ* were transformed into the *L. plantarum* WCFS1 strain by Eppendorf Electroporator 2510 according to Dan *et al*. (2014) [25] with modification. The 100 μl of *L. plantarum* competent cells were mixed with 20 μl of recombinant plasmids and then electroporated at 1.75 kV. After the pulse application, the mixture was inoculated into MRS medium containing 0.5 M sucrose at 37°C for 3 h. Transformed products diluted with 1.0 ml MRS medium were spread on the MRS plates (containing 40 U/ml nisin) and incubated at 30°C for 48 h. The positive transformants were identified by colony PCR using specific primers in Table 1 and were named as LacLM and LacZ recombinants based on carrying plasmids, respectively.

### Protein Purification

The transformants were inoculated into 50 ml MRS medium with 40 U/ml nisin and incubated at 37°C for 48-72 h until the absorbance at 600 nm (OD_600_) reached 3.0. The cultures were centrifuged at 7,552 ×*g* for 10 min at 4°C. The cell pellet was lysed in equal volume of 50 mM phosphate buffer (pH 8.0) by sonication, and then cell lysate was centrifuged at 11,800 ×*g* for 30 min using 100 kD Millipore ultrafiltration centrifuge tube. After that the ultrafiltration liquid was collected and centrifuged again using 10 kD Millipore ultrafiltration centrifuge tube to concentrate the extract of recombinant β-galactosidase. Protein contents were determined by the method of Bradford (1976) [26] using bovine serum albumin as standard.

### The Total β-Galactosidase Activity

Four different strains (including two recombinant strains and the original *L. kefiranofaciens* ATCC51647 and *L. plantarum* WCFS1) were grown in MRS at 37°C for 3 day. 100 ml of fermentation broth was collected and extracted at different OD_600_ level to determin the total β-galactosidase activity. The total β-galactosidase activity was determined using o-nitrophenyl-β-galactopyranoside (ONPG) as the substrate [17]. A 200 μl aliquot of extract of fermentation broth and 1.0 ml ONPG solution (2.5 g/l) were mixed together in sodium phosphate buffer (pH 7.0) and incubated at 37°C for 30 min. The reaction was stopped by 200 μl of 0.1 M sodium carbonate, and then, the absorbance was assayed at 420 nm. One unit (U) of β-galactosidase activity was defined as the quantity of enzyme that liberates 1 μM of O-nitrophenol from ONPG per minute under the assay conditions [11].

### Hydrolysis of Lactose

To examine the lactose hydrolysis, 10 U/ml (ONPG hydrolytic activity) of recombinant extract were added to the milk. Mixed solution was incubated at 37°C for 24 h, and the amount of residual lactose was determined every 4 h by the HPLC described by Büyükkileci and Harsa (2004) [27].

### Transglycosylation

The β-galactosidase from the two recombinants was used to study the generation of GOS. The time-course of GOS synthesis was monitored by incubating 10 U/ml (ONPG hydrolytic activity) of recombinant extract in 25%(w/v) lactose with 50 mM citrate buffer (pH 5.0) at 45°C for 24 h. Samples were collected at different time points, and the contents of lactose, monosaccharides (galactose and glucose) and GOS were analyzed on the Size Exclusion Chromatography (SEC-HPLC) with a Rezex RSO-01 oligosaccharide Ag^+^ column, eluted with Milli-Q water following the methods from Sander *et al*. (2014) [28].

### Fermentation of the Recombinants

Two type of recombinant strain were grown in MRS broth with varying levels of nisin in a 2 L fermenter at different temperature for 72 h. The optimum temperature was determined by the measuring the optical density at 600 nm (OD_600_) of the fermentation broth at 25°C, 30°C, and 37°C respectively. In addition, nisin with concentration of 0, 10, 20, 40, 100, and 200 U/ml was added to the fermentation broth and the optimal dosage of nisin was determined by analyzing the change of OD_600_ value.

### Statistical Analysis

All the data from the three replicates were expressed as the mean ± SD and analyzed by the LSD’*t*-test at *p* < 0.05 using SPSS software (version 19.0, SPSS Inc., USA) to assess statistical difference between mean values.

## Results

### Gene Cloning and Construction of Food-Grade Expression Vectors

*lacLM* and *lacZ*, encoding a putative LacLM-type and LacZ-type β-galactosidase were cloned from *L. kefiranofaciens* ATCC51647, respectively, and inserted into lactobacillal expression vector pMG36e with erythromycin resistant marker. For practical application, food-grade pMG36n-lacs recombinant plasmids were constructed by nisin selective marker *nisI* replacing erythromycin selective marker of pMG36e-*lacs* (Fig. 1). *L. plantarum* overexpressing pMG36n-lacs could grow normally in MRS medium with nisin while control strain could not survive in MRS medium with nisin (Data are not shown). It was preliminarily proved that food-grade Expression system was successfully constructed. The sequencing results verified these constructions and showed that the inserted fragments displayed 100% identity to *lacLM* and *lacZ* genes of *L. kefiranofaciens*. Moreover, the *lacLM* (2,833 bp) encoded a protein of 944 amino acids with a calculated molecular mass of 110 kDa, and it was composed of two overlapping genes, *lacL* (1,884 bp) and *lacM* (960 bp), which encoded large and small subunits with a predicted molecular mass of 73,620 and 35,682 Da, respectively. In addition, the *lacM* gene was detected downstream of *lacL*, and the overlap of two genes was 17 bp. The *lacZ* (2,007 bp) only encoded a polypeptide chain of 668 amino acids with a calculated molecular mass of 76,428 Da. These sequences were consistent with previous findings [17].

### Expression of β-Galactosidase Gene in *L. plantarum*

**Fig. 2:**
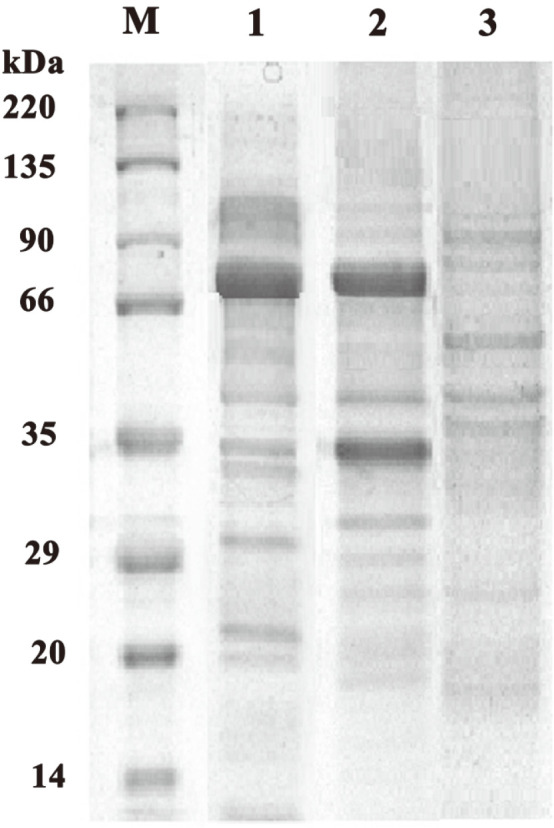
SDS-PAGE analysis of the protein expressed in transformation. Lane M, low-molecular-weight standard; Lane 1, purified protein of recombinant LacZ; Lane 2, purified protein of recombinant LacLM; Lane 3, whole-cell lysates of *L. plantarum* WCSF1 containing empty vector.

SDS-PAGE analysis showed that compared with the original strain, the extracts from LacZ and LacLM recombinants obviously displayed a single band and two bands on SDS-PAGE, respectively, and these bands were all consistent with the expected molecular mass of β-galactosidase in *L. kefiranofaciens* with similar amount of LacZ and LacLM (Fig. 2).

### Enzymatic Properties of the Two β-Galactosidases

The activities of β-galactosidase in the extract from *L. plantarum* WCFS1, original *L. kefiranofaciens* ATCC51647, LacZ and LacLM recombinant strains for hydrolyzing ONPG were investigated. The results showed the hydrolysis effect of β-galactosidase was markedly enhanced with the increase of OD_600_ level in all the four strains, moreover, the rate of increment was larger before OD600=3.0 due to strains in the logarithmic phase (Table 2). After logarithmic phase, the β-galactosidase activity were saturated, and the activity remained unchanged even if the number of cells continued to increase. In addition, at the same OD_600_ value, the rank of β-galactosidase activity was recombinant LacLM > recombinant LacZ > the original *L. kefiranofaciens* > the original *L. plantarum* (Table 2). For example, at OD_600_ 3.0, the β-galactosidase activity from LacLM recombinant reached 7.59 U/mg protein which was 2.2 times of that from LacZ recombinant (3.48 U/mg protein), and 14.6 times of that from the control *L. kefiranofaciens* strain (0.52 U/mg of protein) (Table 2).

**Fig. 3:**
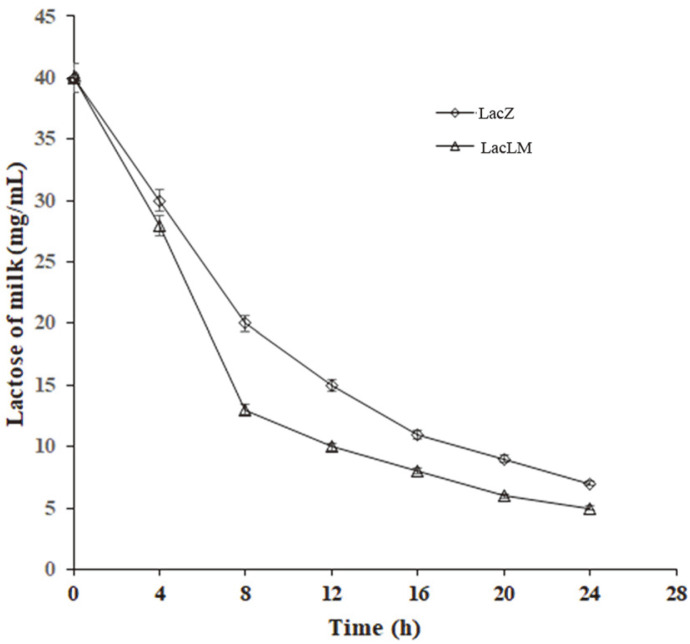
The hydrolysis effect of recombinant β-galactosidase for milk lactose.

Due to the low β-galactosidase activity of the original *L. kefiranofaciens* strain, it was difficult to prepare a high concentration of enzyme solution. Therefore, only two recombinant strains with high β-galactosidase activity were chosen to study the catalytic characteristics of enzymes. Firstly, β-galactosidase crude extracts from the two recombinant strains were added to the milk, and the residual lactose content was measured in milk every 4 h by HPLC. The results showed that more than 87.5% of lactose was hydrolyzed in the two recombinant extracts within 24 h (Fig. 3). Moreover, within 0-4 h, more than 25% of lactose was hydrolyzed in both recombinant extracts with no difference. More than 80% of lactose was hydrolyzed in the LacLM recombinant at 8 h, while the same amount of lactose was hydrolyzed in LacZ till about 16 h. Therefore, although two types of β-galactosidase have the same effect of hydrolysis at beginning, a hydrolysis effect of LacLM-type β-galactosidase then performed better than that of LacZ-type β-galactosidase.

**Fig. 4:**
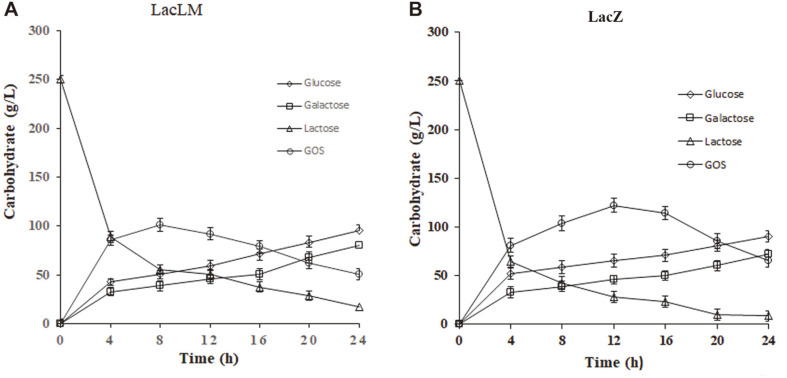
The production of GOS, lactose, glucose and glactose in the course of formation of GOS by recombinant β-galactosidase. (**A**) LacLM; (**B**) LacZ.

Based on the GOS synthesis furtherly, the transgalactosylation activity of β-galactosidase extracts from LacLM and LacZ recombinant strains was assayed in 25% lactose solution. Fig. 4 showed that the content changes of glucose, galactose, lactose and GOS, respectively. With increase of lactose hydrolysis, the amount of glucose and galactose rose gradually in both reaction solution. Furthermore, GOS was synthesized from galactose for the transgalactosylation of β-galactosidase, and the GOS amount reached maximum level at 8 h from LacLM recombinant and 12 h from LacZ recombinant. After that, GOS content decreased, because a part of the GOS might be hydrolyzed again by LacLM and LacZ, respectively [28]. Moreover, within 8-20 h GOS net content was higher in solution LacZ than LacLM, especially at 12 h, which GOS content was 20% noticeably greater in recombinant LacZ than LacLM. In addition, the change pattern of lactose content was different to glucose, galactose and GOS in solution. As a substrate, the lactose amount declined sharply in the first 4 h of reaction process, after that it declined slowly in both extracts.

### Fermentation Temperature and Nisin Addition

**Fig. 5:**
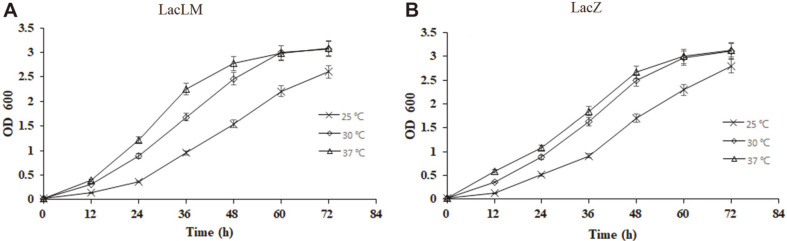
Effect of temperature on the growth of *L. plantarum*. (**A**) *L. plantarum*/pMG36n-*lacLM* (**B**) *L. plantarum*/ pMG36n-*lacZ*.

Under the three fermentation temperatures, the growth trend of the two recombinants was similar. The biomass of both LacLM and LacZ strains at the 37°C and 30°C was higher than 25°C within 72 h, and the biomass of both strains at 37°C was higher than 30°C within 48 h. But after 60 h, there was little difference between 37°C and 30°C, and the OD600 both exceed 3.0 at 72 h (Fig. 5).

**Fig. 6:**
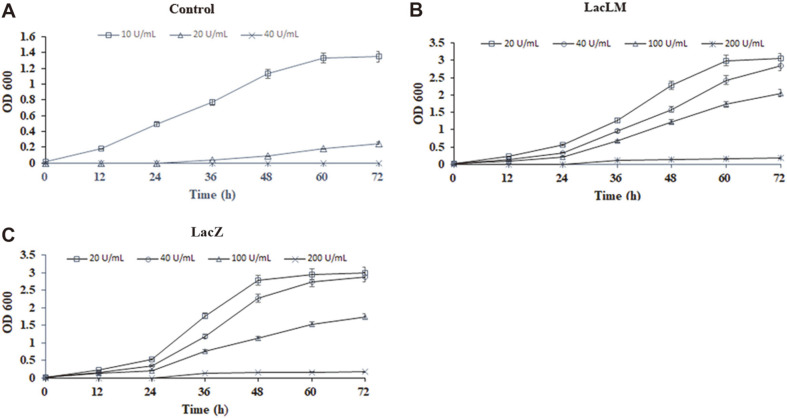
Effect of nisin addition on the growth of *L. plantarum*. (**A**) Original *L. plantarum* (**B**) *L. plantarum*/pMG36n*lacLM* (**C**) *L. plantarum* /pMG36n-*lacZ*.

The effect of nisin on the *L. plantarum* WCFS1was obvious. As can be seen from Fig. 6, the control *L. plantarum* could only tolerate the nisin with a concentration of 10 U/ml and almost stop growing at 20 U/ml. The recombinant *L. plantarum* grew normally at concentrations of 20 U/ml and 40 U/ml, but at 100 U/ml the growth rate dropped significantly, and at 200 U/ml it almost stopped growing.

## Discussion

Lactic acid bacteria (LAB) are widely used in food fermentations and are becoming very attractive microbial cell factories as they can be used for food-related applications or for the production of food relevant enzymes [29, 30]. Moreover, for food applications, expression systems based on food-grade microorganisms having the “generally recognized as safe” (GRAS) status are of considerable interest. Several expression vector for LAB have been available such as pSIP403 and pMG36e [31, 32]. Here, pMG36e and *L. plantarum* WCFS1 were chosen as β-galactosidase overexpression system (Fig. 1). The expression vector pMG36e constructed for lactococci at first, has been reported to be functional in lactobacilli [33]. pMG36e uses erythromycin resistant gene as selective marker for the expression system, however, antibiotic resistant genes which might be transferred to other microorganisms when the producing organism remains in the final product, and the use of antibiotics in fermentations is also be avoided in these food-grade approaches [34]. In contrast, nisin is a small peptide with antimicrobial activity against a wide range of bacteria, produced by lactococci [35] and was approved by the Joint Food and Agriculture Organization/World Health Organization (FAO/WHO) as a safe food additive [36, 37]. So, erythromycin resistant gene of pMG36e-*lacs* was replaced with nisin resistance gene *nisI* using homologous recombinant (Fig. 1). The resulting constructs pMG36n-lacs can inhibit the pore formation activity of nisin to host strain *L. plantarum* WCFS1 and are of high interest for food-application purposes.

β-Galactosidases (lactases, E.C. 3.2.1.23) possess four glycoside hydrolase (GH) families. Among them, GH42 β-galactosidases (LacZ) are prevalent in strains of leuconostoc and lactobacilli which also harbour a GH2 LacLM β-galactosidase [38]. Moreover, *lacLM* have been isolated from different lactobacillus such as *L. fermentum*, *L. sakei*, and *L. coryniformis* [39, 40] and heterologously expressed in different host bacteria to analyze the activity for application. However, LacZ from LAB has few reports in application characteristic. In this study, two β-galactosidase genes *lacLM* and *lacZ* were isolated from *L. kefiranofaciens* ATCC51647 and homologously overexpressed in *L. plantarum* WCFS1, respectively with similar expression level (Fig. 2). Using the ONPG and lactose as substrates, the hydrolytic activity of crude extract from *lacLM* recombinant was higher than that of *lacZ* recombinant (Table 2, Fig. 3), suggesting that like those of other LAB, LacLM type β-galactosidase performed better than LacZ type in hydrolytic activity with different expression levels [18].

The GOS was synthesized from galactose with lactose hydrolysis in 25% lactose solution (Fig. 4) under hydrolysis and transgalactosylation activity of β-galactosidase. In the first 8 hours the increase rate of GOS concentration was similar in both extract of LacLM and LacZ. However, GOS concentration gradually decreased after 8 h in LacLM and 12 h in LacZ, respectively, and was kept higher in LacZ than that of LacLM (Fig. 4). Correspondingly, because a part of lactose was involved in the synthesis of GOS, the amount of lactose remaining in LacZ was lower than LacLM during this period [14, 28]. It is proposed that as the GOS concentration increased to certain degree, a part of the GOS might be hydrolyzed by LacLM and LacZ. As a result, the GOS concentration gradually dropped till GOS synthesis and hydrolysis was be in dynamic equilibrium [28]. Moreover, the point of dynamic equilibrium might be different between LacLM and LacZ as result of different hydrolytic activity (Table 2). Certainly, it was not ruled out that LacLM and LacZ had different transgalactosylation activity. Consequently, it is indicated that LacLM and LacZ may play different roles in carbohydrate metabolism of *L. kefiranofaciens*, LacLM focuses on lactose hydrolysis while LacZ is conducive to promoting GOS synthesis. Therefore, we suggested that *L. plantarum*/pMG36n-*lacLM* will be more suitable for reducing lactose in milk, and *L. plantarum*/pMG36n-*lacZ* will be suitable for GOS production in dairy industry .

The main factors affecting fermentation are temperature, pH, oxygen content and medium composition. As *L. plantarum* was facultative aerobe microorganisms, no extra oxygen supply required during fermentation. Furthermore, due to the production of lactic acid in the progress of fermentation, the pH should constantly decrease. Therefore, two parameters, temperature and nisin addition in the medium were selected as the preliminary exploration of fermentation conditions in this study (Figs. 5 and 6). And the highest growth rate of all the strains was found at 30°C and 37°C (Fig. 5). In addition, 20 and 40 U/ml nisin could be used as selective pressure to keep the stability of expressing vector in recombinants (Fig. 6). Given fermentation cost, 37°C and 20 U/ml of nisin could be chosen as the appropriate condition.

## Figures and Tables

**Table 1:** Sequences of the primers used in this study.

Primer Name	Primer sequences (5’→3’)
LacLM F	GAGCTCATGCAAGCAAATATTAAATGG
LacLM R	AAGCTTTTAAAACTGGTTTAAGATG
LacZ F	GAGCTCATGACACAAACTTTAACACGC
LacZ R	AAGCTTCTATTTAACCAAAACTTGCAC
NisI F	CCAAATTAAAGAGGGTTATAATGAGAAGATATTTAATACTTATTGTGGCC
NisI R	CAGTTTATGCATCCCTTAACCTAGTTTCCTACCTTCGTTGCAAG
Pmg36 F	TATAACCCTCTTTAATTTGGTTATATG
Pmg36 R	GTTAAGGGATGCATAAACTGCATC

*Restriction sites are underlined; F denotes forward primers, R denotes reverse primers; LacLM stand for β-galactosidase LacLM, LacZ for β-galactosidase LacZ, NisI for nisin resistant gene *nis I* and Pmg36 for the linear fragments without erythromycin resistant gene of pMG36e.

**Table 2:** The total activities of β-galactosidase in different OD600 levels.

OD600	The activities of β-galactosidase (U /mg protein)			

	*L. plantarum*	*L. kefiranofaciens*	*L. plantarum* /pMG36n-*lacLM*	*L. plantarum* /pMG36n-*lacZ*
1.0	0.17±0.02	0.24±0.03	1.15±0.16	0.48±0.06
2.0	0.31±0.03	0.39±0.05	2.08±0.24	0.95±0.09
3.0	0.45±0.02	0.52±0.04	7.59±0.57	3.48±0.16
4.0	0.46±0.03	0.58±0.04	7.61±0.71	3.51±0.19
5.0	0.47±0.03	0.59±0.05	7.62±0.68	3.52±0.17

## References

[1] Xingxing W, Jinzhou X, Yusheng J, Yingjie P, Yongjie W (2018). *Lactobacillus kefiranofaciens*, the sole dominant and stable bacterial species, exhibits distinct morphotypes upon colonization in Tibetan kefir grains. Heliyon.

[2] Cheirsilp B, Suksawang S, Yeesang J, Boonsawang P (2018). Co-production of functional exopolysaccharides and lactic acid by *Lactobacillus kefiranofaciens* originated from fermented milk, kefir. J. Food Sci. Technol..

[3] Chen YP, Hsiao PJ, Hong WS, Dai TY, Chen MJ (2012). *Lactobacillus kefiranofaciens* M1 isolated from milk kefir grains ameliorates experimental colitis in vitro and in vivo. J. Dairy Sci..

[4] Slattery C, Cotter PD, O'Toole PW (2019). Analysis of health benefits conferred by *Lactobacillus* species from Kefir. Nutrients.

[5] Sugawara T, Furuhashi T, Shibata K, Abe M, Kikuchi K, Arai M, Sakamoto K (2019). Fermented product of rice with *Lactobacillus kefiranofaciens* induces anti-aging effects and heat stress tolerance in nematodes via DAF-16. Biosci. Biotechnol. Biochem..

[6] Ye S, Weitao G, Yajing P, Jinju W, Ping X, Yanping W (2019). Supplementation with *Lactobacillus kefiranofaciens* ZW3 from Tibetan Kefir improves depression-like behavior in stressed mice by modulating the gut microbiota. Food Funct..

[7] Dandy Y, Lilis N, Ratih DH, Dase H (2020). In vitro characterization of lactic acidbacteria from indonesian kefir grains as probiotics with cholesterol-lowering effect. J. Microbiol. Biotechnol..

[8] Duarte LS, Schöffer JN, Lorenzoni ASG, Rodrigues RC, Rodrigues E, Hertz PF (2017). A new bioprocess for the production of prebiotic lactosucrose by an immobilized β-galactosidase. Process. Biochem..

[9] Ustok FI, Tari C, Harsa S (2010). Biochemical and thermal properties of β-galactosidase enzymes produced by artisanal yoghurt cultures. Food Chem..

[10] Harrington LK, Mayberry JF (2008). A re-appraisal of lactose intolerance. Int. J. Clin. Pract.

[11] Sara CS, Eugénia AM, José AT, Lígia RR (2018). New β-galactosidase producers with potential for prebiotic synthesis. Bioresour. Technol..

[12] Cardoso BB, Silvério SC, Abrunhosa L, Teixeira JA, Rodrigues LR (2017). β-Galactosidase from *Aspergillus lacticoffeatus*: a promising biocatalyst for the synthesis of novel prebiotics. Int. J. Food Microbiol..

[13] Pereira RÁ, Fernández LR, González SMI, Cerdán ME, Becerra M, Sanz AJ (2012). Structural basis of specificity in tetrameric *Kluyveromyces lactis* β-galactosidase. J. Struct. Biol..

[14] Vera C, Guerrero C, Illanes A (2011). Determination of the transgalactosylation activity of *Aspergillus oryzae* β-galactosidase: effect of pH, temperature, and galactose and glucose concentrations. Carbohydr. Res..

[15] Pawlak SA, Marta W, Tomasz PA, Kur J (2014). A novel cold-active β-D-galactosidase with transglycosylation activity from the Antarctic *Arthrobacter* sp. 32cB: gene cloning, purification and characterization. Process Biochem.

[16] Nguyen TH, Splechtna B, Yamabhai M, Haltrich D, Peterbauer C (2007). Cloning and expression of the β-galactosidase genes from *Lactobacillus reuteri* in *Escherichia coli*. J. Biotechnol..

[17] Xi H, Ning H, Yanping W (2016). Cloning, purification and characterization of a heterodimeric β-galactosidase from *Lactobacillus kefiranofaciens* ZW3. J. Microbiol. Biotechnol..

[18] Clarissa S, Kim IS, Michael GG (2010). Heterologous expression of glycoside hydrolase family 2 and 42 β-galactosidases of lactic acid bacteria in *Lactococcus* lactis. Syst. Appl. Microbiol..

[19] Nguyen TH, Splechtna B, Krasteva S, Kneife W, Kulbe KD, Divne C (2007). Characterization and molecular cloning of a heterodimeric β-galactosidase from the probiotic strain *Lactobacillus acidophilus* R22. FEMS. Microbiol. Lett..

[20] Nguyen TT, Nguyen HM, Geiger B, Mathiesen G, Eijsink VG, Peterbaue CK (2015). Heterologous expression of a recombinant lactobacillal β-galactosidase in *Lactobacillus plantarum*: effect of different parameters on the sakacin P-based expression system. Microb. Cell Fact..

[21] Shin JM, Gwak JW, Kamarajan P, Fenno JC, Rickard AH, Kapila YL (2016). Biomedical applications of nisin. J. Appl. Microbiol..

[22] Maischberger T, Mierau I, Peterbauer CK, Hugenholtz J, Haltrich D (2010). High-level expression of *Lactobacillus* β-galactosidases in *Lactococcus lactis* using the food-grade, nisin-controlled expression system NICE. J. Agric. Food Chem..

[23] van de Guchte M, van der Vossen JM, Kok J, Venema G (1989). Construction of a lactococcal expression vector: expression of hen egg white lysozyme in *Lactococcus lactis* subsp. *lactis*. Appl. Environ. Microbiol..

[24] van der Vossen JM, van der Lelie D, Venema G (1987). Isolation and characterization of *Streptococcus cremoris* Wg2-specific promoters. Appl. Environ. Microbiol..

[25] Dan X, Ri N, Jiufeng G, Teng L (2014). Study on the construction of recombined plasmid pMG36e-lacc1 and the electroporation of *Lactobacillus buchneri*. Biomed. Mater. Eng..

[26] Bradford MM (1976). A rapid and sensitive method for the quantitation of microgram quantities of protein utilizing the principle of protein-dye binding. Anal. Biochem..

[27] Büyükkileci AO, Harsa S (2004). Batch production of L (+)-lactic acid from whey by *Lactobacillus casei* (NRRL B-441). J. Chem. Technol. Biotechnol..

[28] Sander SVL, Bas JHK, Lubber D, Johannis PK (2014). ^1^H NMR analysis of the lactose/β-galactosidase-derived galactooligosaccharide components of Vivinal GOS up to DP5. Carbohydr. Res..

[29] Aaron G, Geoff WS, Andrew RB, Sandra EK, Sally LG (2010). Recent advances refining galactooligosaccharide production from lactose. Food. Chem..

[30] Collins JK, Thornton G, Sullivan GO (1998). Selection of probiotic strains for human applications. Int. Dairy. J..

[31] Fulu L, Yating Z, Wanjin Q, Duolong Z, Haijin X, Per EJS (2019). Restructured *Lactococcus lactis* strains with emergent properties constructed by a novel highly efficient screening system. Microb. Cell. Fact..

[32] Intaratrakul K, Nitisinprasert S, Nguyen TH, Haltrich D, Keawsompong S (2017). Secretory expression of β-mannanase from Bacillus Circulans NT 6.7 in *Lactobacillus plantarum*. Protein. Expr. Purif..

[33] Yuelan Z, Lufeng J, Teng L, Min W, Wenbo C, Yongzhan B (2015). Construction and immunogenicity of the recombinant *Lactobacillus acidophilus* pMG36e-E0-LA-5 of bovine viral diarrhea virus. J. Virol. Methods..

[34] Landete JM (2016). A review of food-grade vectors in lactic acid bacteria: from the laboratory to their application. Crit. Rev. Biotechnol..

[35] Willem MV (1999). Safe and sustainable systems for food - grade fermentations by genetically modified lactic acid bacteria. Int. Dairy J..

[36] Jae MS, Ji WG, Pachiyappan K, Christopher F, Alexander HR, Yvonne LK (2016). Biomedical applications of nisin. J. Appl. Microbiol..

[37] Cotter PD, Hill C, Ross RP (2005). Bacteriocins: developing innate immunity for food. Nat. Rev. Microbiol..

[38] Qu P, Junmin Z, Lina L, Yanguang C, Fuquan H, Jinchuan L (2010). Functional identification of a putative β-galactosidase gene in the special lac gene cluster of *Lactobacillus acidophilus*. Currt. Microbiol..

[39] Corral JM, Bañuelos O, Adrio JL, Velasco J (2006). Cloning and characterization of a β-galactosidase encoding region in *Lactobacillus coryniformis* CECT 5711. Appl. Microbiol. Biotechnol..

[40] Liu GX, Kong J, Lu WW, Kong WT, Tian H, Tian XY (2011). β-Galactosidase with transgalactosylation activity from *Lactobacillus fermentum* K4. J. Dairy Sci..

